# Precision medicine in the treatment stratification of AML patients: challenges and progress

**DOI:** 10.18632/oncotarget.26492

**Published:** 2018-12-28

**Authors:** Ines Lohse, Kurt Statz-Geary, Shaun P. Brothers, Claes Wahlestedt

**Affiliations:** ^1^ Center for Therapeutic Innovation, Miller School of Medicine, University of Miami, Miami, FL, USA; ^2^ Department of Psychiatry and Behavioral Sciences, Miller School of Medicine, University of Miami, Miami, FL, USA; ^3^ Molecular Therapeutics Shared Resource, Sylvester Comprehensive Cancer Center, University of Miami, FL, USA

**Keywords:** AML, *ex vivo* drug sensitivity screens, omics-based screens, phenotypic screens, precision medicine

## Abstract

Recent advances in high throughput technologies have led to the generation of vast amounts of clinical data and the development of personalized medicine approaches in acute myeloid leukemia (AML). The ability to treat cancer patients based upon their individual molecular characteristics or drug sensitivity profiles is expected to significantly advance cancer treatment and improve the long-term survival of patients with refractory AML, for whom current treatment options are restricted to palliative approaches. The clinical development of omics-based and phenotypic screens, however, is limited by a number of bottlenecks including the generation of cost-effective high-throughput data, data interpretation and integration of multiple approaches, sample availability, clinically relevant timelines, and the development and education of multidisciplinary teams.

Recently, a number of small clinical trials have shown survival benefits in patients treated based on personalized medicine approaches. While these preliminary studies are encouraging, larger trials are needed to evaluate the utility of these technologies in routine clinical settings.

## INTRODUCTION

Increasing incidence rates (3.4% per year over the last 10 years) of acute myeloid leukemia (AML) are a growing concern in an aging population [1, 2]. Disease heterogeneity resulting from variability in leukemic cell maturation state, a large number of genetic aberrations among patients and existence of multiple disease clones within a single patient present a major challenge in AML treatment. While extensive research efforts over the last decade have shed some light on the biology of AML, overall 5-year survival of AML patients has remained low at ~26% [1, 2]) and is specifically low in the population of patients that are above 60 years of age (5–15%) [1, 2]. Significant advances in next generation sequencing technologies have improved understanding of the molecular events that lead to the initiation and propagation of AML. However, until recently, personalized medicine in AML has been limited to characterizing patients into prognosis groups based on karyotypes to guide treatment options. Recent studies suggest that a single AML sample contains around 400 mutations, with 13 of these residing in coding regions [3, 4]. Several potential driver mutations have been described and include NPM1, CEBPA, DNMT3A, TET2, RUNX1, ASXL1, IDH2, and MLL [5]). This molecular information led to the development of more accurate classification systems and provided new targets for minimal residual leukemia monitoring, and drug discovery and development [6]. In large part because of the increasingly apparent heterogeneity of this disease, too few personalized approaches for patients with AML have been established for clinical use. Modern AML therapy remains based on core principles, which include patient selection for induction chemotherapy, timing of stem cell transplantation and optimal supportive care. Therefore, patients often receive the same standard of care treatments: the nucleoside analogue cytarabine along with a topoisomerase II inhibitor such as daunorubicin, a regimen that is associated with significant side effects. In particular, the aggressive nature of the standard of care treatments present major tolerability concerns in older AML patients, a patient population that generally shows rapid disease progression in combination with poor overall health and low tolerance of systemic anti-cancer treatments [2]. Hence, no satisfactory standard treatment exists for this population and most of the patients are physically unable to tolerate aggressive chemotherapy [2]. Therefore emerging approaches in personalized medicine may be key to improving patient outcomes but have been difficult to establish for clinical use due to high costs and long turnover times.

### Omics profiling

The most common form of personalized medicine approaches in the context of AML is the use of genomic molecular profiling in order to define molecular subtypes then select the most appropriate therapy. The development of molecular profiling approaches for AML patients such as whole genome sequencing (WGS), whole exome sequencing (WES), and transcriptome sequencing (RNAseq) have generated certain insight into the biology of AML development, progression and treatment resistance. Indeed, new knowledge concerning the heterogeneous and combinatorial driver events of AML is now being translated into clinics in order to improve treatment planning, drug delivery and exploitation of novel cellular targets.

One of the most successful examples of genetic screening in AML is the treatment of acute promyelocytic leukemia (APL). APL is characterized by the t(15;17) cytogenetic abnormality, resulting in PML-RARA fusion gene and the negative inhibition of wild type retinoic acid receptor and myeloid differentiation [7]. Treatment of APL using all-trans-retinoic acid (ATRA) and arsenic trioxide (ATO) have resulted in an increase of cure rates from 30% to 90% [8]. Other examples of successful advances in individualized AML therapies are core binding factor (CBF) AML and FLT3 mutated AML where patient stratification has significantly increased survival in the patient populations [9].

While the advances in molecular profiling have significantly increased the survival of patients by providing a more advanced classification of AML subtypes for treatment stratification, these technologies have made little impact on patients not falling into such specific subtypes. Several studies are currently evaluating the utility of genomics-guided treatment approaches for AML patients that failed standard-of-care or those were aggressive chemotherapy that is commonly used in AML is not possible (Table 1).

**Table 1:** Clinical trials evaluating precision medicine approaches in AML patients

Study name	Status	Center	Study type
High Throughput Drug Sensitivity Assay and Genomics- Guided Treatment of Patients With Relapsed or Refractory Acute Leukemia	recruiting	Single center	interventional
iCare for Cancer Patients	recruiting	Single center	interventional
Biomarkers in Samples From Adult Patients With Acute Myeloid Leukemia Who Failed Existing Standard-of-Care Treatment	unknown	unknown	observational
Molecular Characterization of Acute Erythroid Leukemia (M6-AML) Using Targeted Next-generation Sequencing	completed	Single center	observational
Prospective Study of Molecular Predictors of Survival in Myelodysplastic Syndromes	Not recruiting	Single center	interventional
Biomarker Study of Chemotherapy Resistance and Outcomes in Samples From Older Patients With Acute Myeloid Leukemia	completed	unknown	observational
Accuracy Testing of the Chromosomal Aberration and Gene Mutation Markers of the AMLProfiler	terminated	Multicenter	observational
Genomics-Based Target Therapy for Children With Relapsed or Refractory Malignancy	recruiting	Single center	interventional
Beat AML Core Study	recruiting	Single center	interventional
Diagnostic Platform to Perform Centralized and Standardized Rapid Molecular Diagnosis by Next Generation Sequencing (NGS) in Patients Diagnosed WithAcute Myeloid Leukemia.	recruiting	Multicenter	observational
ChEmo-Genomics Based Treatment of Acute Myeloid Leukemia	recruiting	Single center	interventional
Treatment for Relapsed/Refractory AML Based on a High ThroughputDrug Sensitivity Assay	Not recruiting	Single center	interventional
Personalized Kinase Inhibitor Therapy Combined With Chemotherapy in Treating Patients With Newly Diagnosed Acute Myeloid Leukemia	recruiting	Single center	interventional
Next Generation Sequencing (NGS) in Familial Acute Myeloid Leukemiaand Myelodisplastic Syndromes	recruiting	Single center	observational
Epigenetic Reprogramming in Relapse/Refractory AML	recruiting	Multicenter	interventional
Biomarker Study of Chemotherapy Resistance and Outcomes in Samples From Older Patients With Acute Myeloid Leukemia	completed	Single center	unknown
Phase I Epigenetic Priming Using Decitabine With Induction Chemotherapy in AML	completed	Single center	unknown
A Trial of Epigenetic Priming in Patients With Newly Diagnosed Acute Myeloid Leukemia	recruiting	Single center	interventional
Decitabine, Cytarabine, and Daunorubicin Hydrochloride in Treating Patients with Acute Myeloid Leukemia	Active, Not Recruiting	Multicenter	interventional
Epigenetics, Vitamin C and Abnormal Hematopoiesis – Pilot Study	Recruiting	Single center	interventional
Study of Sensitization of Non-M3 AML Blasts to ATRA by Epigentic Treatment With Tranylcypromine (TCP)	Recruiting	Multicenter	interventional
Decitabine Followed by Idarubicin and Cytarabine in Treating Patients with Relapsed or Refractory AML and MDS	Terminated	Single center	interventional

The majority of AML patients have recurrent mutations or chromosomal rearrangements that can be utilized as predictors of clinical outcome and treatment sensitivity [10–17]. A number of patients, however, present with normal karyotype and/or no targetable mutations. In contrast to the genetic alterations, epigenetic changes resulting in aberrant gene expression are reversible and can be targeted pharmacologically. A number of studies have shown that genes regulating DNA methylation are frequently mutated in AML [18] and that epigenetic alterations contribute to the aggressive phenotype of AML [19–28]. Clinically, epigenetic treatment approaches using decitabine and azacitidine have shown encouraging response rates [29–33] and a number of other compounds and protocols are currently being evaluated in Phase I and II clinical trials (Table 1). As yet, prognostic and predictive molecular biomarkers that enable selection of patients who are likely to benefit from epigenetic therapy are not available for clinical testing. While techniques, such as pyrosequencing, for the DNA methylation analyses exist [32, 33], they are not currently used for patient stratification.

While the current practice of using WGS or WES for the genetic screening of cancers can provide information about the mutational landscape of individual patients, for most patients the screens cannot inform an effective treatment plan because the majority of observed mutations remain non-targetable. Using only genomics-based screening for the molecular profiling of AML patients fails to recognize changes in transcript splicing and the epigenome. Additionally, these screens fail to evaluate changes in signaling pathways or the metabolome, both of which can significantly impact drug sensitivity independent of mutational status. A recent study interrogated 227 phosphoproteins in 650+ cancer cell lines and demonstrated that these data were better at predicting drug response than whole genomic or transcriptomic data [34]. Integrating multiple omics approaches in the development of treatment plans, however, can significantly delay patient treatment due to long turnover times and requires the integration of large datasets which can be difficult in a routine clinical setting. Although omics testing has become much more affordable in the last decade, the use of multiple omics tests and complex data analyses significant increase treatment costs. Also, the heterogeneity of AML presents a significant hurdle that needs to be overcome before genetic testing could guide treatment in all cases.

### Drug sensitivity testing

Rather than attempting to match molecular abnormalities to targeted therapies, drug sensitivity testing (DST) approaches, such as *ex vivo* drug sensitivity screening, use samples of individual patient tumors to evaluate patient-specific drug sensitivity profiles. These assays are especially attractive in AML patients where tumor samples are easily available through peripheral blood draws or bone marrow biopsies and can be evaluated at multiple time points throughout treatment. Drug sensitivity testing is a powerful tool for the treatment stratification of AML patients that has been implemented successfully by different groups [34–39]. By relying on drug sensitivity rather than genetic abnormalities, these screens can provide treatment options in the absence of targetable mutations. Generally, drug sensitivity screen utilize libraries of FDA approved compounds. Treating physicians have therefore access to treatment protocols that have been established for AML or can be adapted based on information obtained in other cancers and negotiations with insurance companies can be based on prior use of these drugs.

In contrast to most omics profiling techniques, phenotypic screens can be performed and evaluated in a relatively short time frame and thus allowing physicians to utilize a precision medicine approach without delaying patient treatment. We have shown that drug sensitivity screens can be performed in a clinical setting with a turnaround time of 10 days [35]. Additionally, retesting of liquid biopsies over the course of treatment cycles may allow the physician to rapidly adapt treatment plans in response to developing or changing drug resistance patterns. This will likely also provide insight into how treatment with specific drugs or classes of drugs such as kinase inhibitors influence resistance to non-related compounds like DNA-damaging agents.

A number of recent studies in AML patients reported survival benefits in response to DST-guided therapy. Staib et al. showed that drug sensitivity screening for daunorubicin and cytarabine in AML patients, had a 94% and 81% predictive accuracy on the treatment outcome of 57 patients [38]. In this study, other drugs displayed lower predictive accuracy: mitoxantrone 53%, idarubicin 66%, fludarabine 50% and thioguanine 22% [38]. In order to avoid the rapid development of treatment resistances in response to single compound treatment, Kurtz et al. focused on testing combination therapies *ex vivo* [37]. They found that combining a proliferation inhibitor such as the CDK4/CDK6 inhibitor palbociclib, with an anti-apoptotic agent like the BCL-2 inhibitor venetolax improves treatment efficacy. While these studies displayed high prediction accuracy and increase treatment efficacy, they used only a limited number of compounds, most of which are already routinely used in AML treatment. They also did not consider non-cytotoxic treatment options.

A pilot study recently performed by our group investigated the utility of DST in patients with refractory AML using a compound library of 215 FDA-approved compounds that included cytotoxic drugs commonly used in the treatment of AML and solid tumors as well as a number of non-cytotoxic compounds [35]. The clinical outcome of DST-guided therapy was compared to physician-directed therapy in 12 AML patients. DST-guided therapy resulted in significant increase in treatment responses when compared to patients receiving physician-guided therapies [35]. This study further emphasized the diversity of AML patients with respect to the response towards a larger panel of anti-cancer agents, where no single compound or class of compounds was effective in all of the tested patients. Additionally, the DST assay generally suggested compounds that were not typically considered by the treating physician, such as gemcitabine. Similar results were observed in a study performed by Pemovska et al. in a cohort of chemorefratory AML patients [36]. There, a patient treated based on DST-guided therapy displayed a significant reduction of bone marrow blasts in response to treatment. Both studies [36, 40] used a version of the drug sensitivity scoring, originally developed by Yadav et al. [34], to assess overall drug responses incorporating information on drug potency, efficacy, effect range and therapeutic index, making it possible to rank compounds over multiple clinically relevant parameters. This computational analysis is aimed at creating an unbiased evaluation of treatment responses and generation of clinically relevant treatment suggestions.

While initial studies using DST appear promising, larger trials are necessary to establish clinical diagnostic utility and treatment efficacy. These studies are hampered by the large cohort sizes necessary to adequately evaluate the utility of such large screening libraries and the development of high throughput screening assays that are able to process samples for a large number of patients in a clinically feasible timeframe. A number of trials are currently investigating drug sensitivity testing in the clinical setting (Table 1).

## CONCLUSIONS

AML treatment largely relies on the use of aggressive chemotherapy aimed to induce cancer remission rather than cure. While this treatment approach is moderately successful in some patients, relapse is frequent and most patients, especially those over 60 years of age, rapidly succumb to the disease [1, 2, 41].

Recent advances in omics technologies have provided unpreceded insight into the biology of AML and also provided numerous novel targets for drug development efforts. A number of studies have demonstrated the extent of genetic variability of AML patients [3–5, 39–41] further emphasizing that the standard *one size fits all* approach can no longer be justified in this disease.

While genetic screening has led to improved clinical outcomes for certain patients, specifically those with APL and CBF AML, the majority of genetic aberrations in AML patients are not targetable with currently available drugs [5, 7, 9–12, 41–43]. Even though WGS or WES are used in a number of clinical trial in AML patients, these methods are rarely used to stratify patients due to long turn over times that delay the treatment start [42–44].

Drug sensitivity testing on the other hand, can be performed without delaying patient treatment due to short assay times and streamlined analysis. Because most screening libraries contain mainly FDA-approved compounds, treatment implementation is simple for the treating physician and can be done in a routine clinical setting. Although a recent set of small studies have shown significant benefits of stratifying patients based on DST results [34–38], better powered studies will be necessary to further develop the platforms for routine clinical use.

In the ASCO (American Society of Oncology) guideline published by Samson et al. (2005) [45], prospective studies comparing assay directed treatment and empiric treatment were critically reviewed and the authors concluded that no benefit was obvious for the former approach. These guidelines were updated in 2011 and the conclusion remained the same [46]. Previous efforts to predict responsiveness to therapy in cancer patients based on a priori laboratory testing, have been limited to the use of conventional chemotherapy drugs for patients with solid tumors [36, 47–57] and hampered by long turn around times that delayed patient treatment. In addition, the *ex-vivo* responses were often non-selective, difficult to interpret, and challenging to translate into clinical practice. Indeed, for most approaches, clinical validation of candidate agents was lacking [47, 48, 50, 53–57]. In many cases, treatment proposed by an assay did not differ from what the clinician would have selected empirically. In the time since the last ASCO review in 2011, significant progress has been made in both the assay technology as well as the analysis of drug sensitivity screening approaches and the majority of the limitations have been addressed. Specifically, the ability to analyze larger compound libraries allows for treatment proposals outside the clinical routine. The development of novel analysis algorithms addressing previous difficulties in cancer selectivity and assay interpretation [34–36]. Cancer selectivity in particular has been addressed by Swords et al. [35] through the use of normal white blood cells as a surrogate for normal tissue toxicity allowing for the selection of agents with high cancer and low normal toxicity thus reducing treatment side effects. The use of these drug sensitivity screens in larger randomized clinical trial will demonstrate feasibility of these approaches in a clinical routine setting.

Both, omics and DST, approaches generate significantly different datasets that are valuable for understanding the biology of AML and stratifying patients for treatment. While DST approaches seems to be clinically more advanced for treatment stratification, patients would benefit from precision medicine approaches combining multiple omics strategies, such as WGS/WES and epigenetic screening, with drug sensitivity testing. A recent study describing the initial findings from the Beat AML program has aimed to integrate whole-genome sequencing, RNA sequencing and drug sensitivity testing in a large cohort of AML patients [58]. This study generated a large dataset using 672 tumor specimens from 562 patients. The integration of mutational status, gene expression and response to a panel of 122 small molecules allows the comparison of specific mutations and drug response, thus providing a powerful tool for drug development [58]. However, the data was not used to stratify patients for treatment and did not address the clinical utility of either of the evaluated methods [58]. By combining these unique datasets for patient stratification in a clinical setting, we may improve the survival rates for today's AML patients and gain further insight into the molecular and genetic diversity of this complex disease.
